# Temperature, but Not Available Energy, Affects the Expression of a Sexually Selected Ultraviolet (UV) Colour Trait in Male European Green Lizards

**DOI:** 10.1371/journal.pone.0034359

**Published:** 2012-03-27

**Authors:** Katalin Bajer, Orsolya Molnár, János Török, Gábor Herczeg

**Affiliations:** 1 Behavioural Ecology Group, Department of Systematic Zoology and Ecology, Eötvös Loránd University, Budapest, Hungary; 2 Ecological Genetics Research Unit, Department of Biosciences, University of Helsinki, Helsinki, Finland; Macquarie University, Australia

## Abstract

**Background:**

Colour signals are widely used in intraspecific communication and often linked to individual fitness. The development of some pigment-based (e.g. carotenoids) colours is often environment-dependent and costly for the signaller, however, for structural colours (e.g. ultraviolet [UV]) this topic is poorly understood, especially in terrestrial ectothermic vertebrates.

**Methodology/Principal Findings:**

In a factorial experiment, we studied how available energy and time at elevated body temperature affects the annual expression of the nuptial throat colour patch in male European green lizards (*Lacerta viridis*) after hibernation and before mating season. In this species, there is a female preference for males with high throat UV reflectance, and males with high UV reflectance are more likely to win fights. We found that (i) while food shortage decreased lizards' body condition, it did not affect colour development, and (ii) the available time for maintaining high body temperature affected the development of UV colour without affecting body condition or other colour traits.

**Conclusions/Significance:**

Our results demonstrate that the expression of a sexually selected structural colour signal depends on the time at elevated body temperature affecting physiological performance but not on available energy gained from food *per se* in an ectothermic vertebrate. We suggest that the effect of high ambient temperature on UV colour in male *L. viridis* makes it an honest signal, because success in acquiring thermally favourable territories and/or effective behavioural thermoregulation can both be linked to individual quality.

## Introduction

Animals use a diverse range of behavioural displays, chemical cues and morphological traits to signal phenotypic condition, or social or reproductive status [1]–[4]. For instance, bright plumage or colourful ornaments of the integument may function as sexual signals [1], [5], [6]. Costly sexual signals can reliably advertise individual quality through their production and/or maintenance cost [7], [8]. Hence, traits limited by environmental constraints often act as honest signals in intra- or intersexual communication [9].

Animals produce colours through two fundamental mechanisms (in this paper, we do not discuss bioluminescence or fluorescence): (i) light absorbing pigment molecules (hereafter pigment-based colours) or (ii) light scattering microstructures in the layers of dermal tissue or plumage (hereafter structural colours) [10], [11]. Pigment-based colours are produced by pigment molecules (e.g. yellow and red caused by pteridins and carotenoids, and brown caused by melanin). Developmental and maintenance costs of some pigment-based colours (e.g. carotenoid or melanin-based colours) are well explored in a range of model taxa, mainly fishes and birds [9], [12]–[15]. Structural colours rely on a wide range of different nanoscale structures, including thin-film multilayer reflectors [11], [16], [17], different types of surface gratings (diffraction gratings, Bragg gratings) [18], nanosphere arrays [19] and collagen arrays [20], [21]. Pigments may also interact with the integument structures, resulting in new colours (e.g. green) [11], [22]. In the great majority of cases, colours such as iridescent, blue and ultraviolet [UV] are produced by light scattering and/or reflective structures, however, there are several exceptions [23], [24]. Therefore, structural colour was first rejected as a signal of quality, because it was regarded as cheap to produce compared to colours based on costly pigments [21]. However, recent studies suggest that developing and maintaining structural colouration might be just as costly as pigment-based colouration [25]. Reduced intensity of structural colours due to food stress or parasite infection and the condition-dependence of certain structural colours have been reported in different bird species [26], [27] and positive relationship between structural colour and immune response intensity was reported in birds and lizards [28], [29]. Further, structural colours of some invertebrates showed nutritional condition-dependence [30]–[32]. However, manipulative experiments testing for the environmental effects on expression of structural colours are still scarce.

Within the topic of sexual selection [1], reptiles have been neglected compared to other vertebrates (birds or mammals) despite their number of colourful ornaments, chemical signals and behavioural displays [29], [33]. Among structural colours, UV signals are used in male competition [29], [34]–[36] and in both male and female mate choice [37], [38] in lizards. Moreover, several lizard species show substantial sexual dichromatism in the UV range [39], [40]. However, there is only limited information available on the development of reptiles' colouration. We have data on how colours are determined in reptilian integument [11], but the costs of the mechanisms underlying colour expression remain poorly understood.

In ectotherms, another major challenge besides gathering the available energy or special nutrients needed is achieving proper body temperature. Temperature-dependent kinetics of biochemical reactions (Q_10_ effect) determines the body temperature at which metabolism is optimal [41]. Therefore, ectotherms have to keep their body temperature in an optimal range to enhance their metabolism and maximize their physiological performance by thermoregulation [42], [43]. Small ectotherms regulate their body temperatures almost exclusively by behavioural thermoregulation [44], [45], which has several costs including the time and energy invested into the behaviour, the lost opportunities for foraging and mating, and increased predation risk [46].

There is no information available about how environmental effects might influence the expression of structural colouration in reptiles. However, because all important aspects of physiological performance are body temperature dependent in ectotherms [43], reptiles make excellent models to study the effect of temperature on structural colour development. Further, the effects of available energy and physiological performance can be easily disentangled by manipulating food supply and environmental temperature.

Female European green lizards, *Lacerta viridis*, prefer males with high throat UV brightness and chroma [38], and males with higher UV chroma are more likely to win male-male fights [36]. Hence, UV colour of the throats of male *L. viridis* is likely to be under strong sexual selection. In the present study, we investigated how certain environmental conditions (i.e. available food and ambient temperature) affected the annual development of the nuptial throat colour patch of male *L. viridis*. Taking advantage of the fact that lizards are ectothermic animals, we could uncouple the effects of available energy and the possibility to utilize that energy (i.e. physiological performance). To do that, we conducted a factorial experiment manipulating food levels and the time during which lizards were able to achieve high body temperature.

## Methods

### Ethics statement

Experiments were performed according to the guidelines of the Hungarian Act of Animal Care and Experimentation (1998, XXVIII, section 243/1998), which conforms to the regulation of animal experiments by the European Union. The experiment was done under the license of the Middle-Danube-Valley Inspectorate for Environmental Protection, Nature Conservation and Water Management (no. 31870-3/2009). The animals did not show any signs of health problems or injuries during the experimental period and were released to their initial capturing location in the field at the end of experiment.

### Study animals

The European green lizard is a medium sized lacertid lizard (snout-vent-length [SVL] = 80–120 mm) which occupies diverse habitats over a wide range in Europe [47]. In our population, males emerge from hibernation in April; females become active 1–2 weeks later than males. Mating season starts in May and lasts till the first half of July during which males have (to the human eye) blue nuptial colouration on their throats. The throat patch has a strong UV component [36], [38]. Nuptial colouration appears on males' throats shortly after emergence and becomes more and more intensive during the next five to seven weeks. After the mating season, nuptial colouration fades, disappearing by the end of summer.

We captured 60 adult male *L. viridis* at the end of April 2009, right after they terminated hibernation and before the onset of mating season, by noosing. Our study area is a forest-scrubland mosaic segmented with dry grasslands near Tápiószentmárton, Hungary (47°20′25″N, 19°47′11″E). All individuals were captured within four days. After capture, they were weighed with a digital scale and their snout-vent length (SVL) was measured with a digital caliper. The lizards were housed individually in plastic boxes (size: 60 cm×40 cm 30 cm, length, width, height, respectively) at our field station 2 km from the capture site. Before the onset of the experiments (1 to 4 days period) they were fed with crickets (*Gryllus domesticus*) and mealworms (*Tenebrio molitor*) dusted with vitamin powder, and water was provided *ad libitum*.

### Spectrometry of colouration

We measured the reflection of the lizards' throats with a spectrometer type Ocean Optics 2000 [35], complete with a Mini-D2 deuterium-halogen lamp and a R700-4 bifurcated fiber-optic fiber (Ocean Optics Inc., Dunedin, Florida). The single ending of the probe was fixed into an RPH1 holder (Ocean Optics Inc., Dunedin, Florida), avoiding all possible light from the environment to influence our measurement. The illuminated area was 6 mm in diameter and it was a constant 3 mm distance and 90° angle with the surface. To get a representative sample of the uneven throat colouration [48], we made three consecutive readings on random spots of the ventral side of the throat patch, with the probe removed between each reading. For the analyses, we used the mean of the three measurements. Reflectance was calculated relative to WS-1 Diffuse Reflectance Standard as a white standard (reflectivity: >98% at 250–1500 nm wavelengths) using the SpectraSuite software (Ocean Optics, Inc.) [34]. Every individual was measured twice, one day before (initial colouration) and immediately after the experimental period (final colouration). Body weight was also re-measured at the end of the experiment (see below). Measurements were taken across the spectrum of 320–700 nm wavelengths. As we are not aware of the visual system of *L. viridis*, we used this as the broadest range of wavelengths known to be visible to lizards [49]. White reference was standardized between each individual, and dark reference ( = no incoming light) was also re-measured periodically to avoid problems with spectrophotometer ‘drift’ [48]. We calculated five variables describing throat colour [38]: (1) brightness, the total reflectance from 320 and 700 nm, (2) UV brightness, the total reflectance from 320 and 400 nm, (3) UV chroma (relative UV intensity), the percent of reflectance measured in the UV range compared to total reflectance (R_320–400_/R_320–700_), (4) blue brightness, the total reflectance from 400 and 490 nm, and (5) blue chroma (relative blue intensity), the percent of reflectance in the blue range compared to total reflectance (R_400–490_/R_320–700_). Similarly to our previous papers [36], [38], we used the range between 320 and 400 nm (UV by definition) as a separate trait, because a visual inspection of the reflectance curve revealed that there is a reflectance peak in the UV range that drops drastically around 410 nm [36], [38], which is very close to the end of the UV range (400 nm). However, we also ran our statistical analyses (see below) using the range from 320 to 410 nm as a separate trait to better follow the reflectance curve of male *L. viridis* during reproduction. The analyses gave the qualitatively similar results, so we report only the original results for the sake of consistency with our earlier studies.

### Experimental setup

We placed lizards in individual plastic boxes (size: 60 cm×40 cm 30 cm, length, width, height, respectively). The boxes were illuminated with Repti Glo 2.0 Full Spectrum Terrarium Lamps (Exo Terra, Rolf C. Hagen Inc., Holm, Germany), which radiates minimal heat, and the photoperiod was held natural (14L: 10D). We used heating cables placed underneath the boxes to ensure diffuse high temperature in the whole area of the box. When heating was turned off, temperature in the boxes decreased considerably (see below). We created four treatment groups with 15 randomly chosen male lizards in each. Food and temperature treatments were applied in a factorial design. The experiment lasted 30 days, from 28^th^ April to 27^th^ May.

### Food and temperature treatments

We applied two food treatments. In the “high food” treatment, lizards were offered 10 ml (Mean = 5 g, Standard Deviation [SD] = 0.04 based on 20 trials) mealworms dusted with vitamin powder three times a day (8:00h, 11:00h, and 14:00 h). Lizards could not consume that food completely, so we removed mealworms that were not eaten (an average of 10 ml mealworms remained) by lizards at the end of the day (17:00h). Therefore, we assume that every individual was able to obtain its optimal amount of food (i.e. *ad libitum*). In the “low food” treatment, 2 ml (Mean = 1 g, SD = 0.03 based on 20 trials) of mealworms dusted with vitamin powder were given three times a day, in the same time schedule as in the optimal food treatment (see above). Lizards consumed all mealworms and kept on searching for more, therefore we assumed that this amount of food was not optimal for them. We also applied two temperature treatments: in the “high temperature” treatment the heating system was switched on for 10 hours a day (07:00 h–17:00 h) while in the “low temperature” treatment only for five hours (07:00 h–12:00 h). When the heater was on, mean temperature in the boxes was 29.2°C (SD = 0.41, measured in all boxes) which is within the range of selected body temperature of *L. viridis* in the beginning of the annual activity season (22.5–33.8°C) [50]. When the heater was off, the temperature dropped considerably, to 17°C (SD = 0.42, measured in all boxes), which is lower than minimum selected body temperature (see above). We assume that lizards' physiological performance was much higher during the heated than during the non-heated period, as they were able to achieve body temperature closely to their optima [43]. Photoperiod was held constant in all treatment groups (see above). The treatments were assigned randomly for every individual. Water was provided *ad libitum* for all treatment groups.

### Statistical analyses

We applied General Linear Models (GLMs) with the change (measure after the experiment – measure before the experiment) in body weight, UV chroma, UV brightness, blue chroma, blue brightness, and total brightness as dependent variables, SVL as a covariate, and treatments (food and temperature) as fixed factors. We also included the interaction between the two factors in the models. Alternative models were also tested which were identical to those above except that the two measures (before/after treatment) of the individuals were entered as repeated measures. These models revealed exactly the same patterns (data not shown), so we only report results from the first GLMs. All statistics were computed using Statistica for Windows v. 10.0 (Statsoft Inc., Tulsa, OK, USA).

## Results

**Figure 1:**
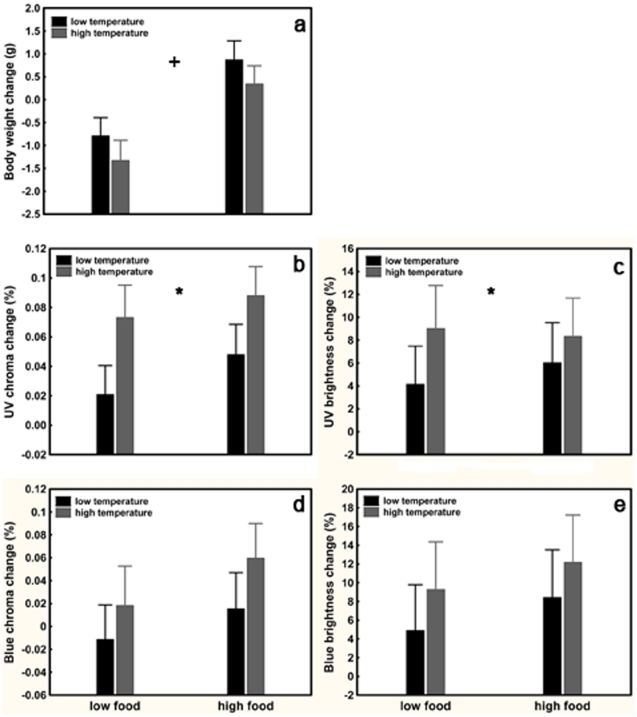
Effects of food and temperature treatments on the body weight change (a); relative (b; UV chroma) and absolute UV reflectance (c; UV brightness); relative (d; blue chroma) and absolute blue reflectance (e; blue brightness) change of the throat nuptial colour patch of male *Lacerta viridis* during the 30 day experimental period. The sign ‘+’ denotes significant food treatment, while ‘*’ significant temperature treatment effects. Least Squares means+95% Confidence Intervals are shown.

GLMs revealed a significant effect of the food treatment on body weight change (Table 1). Food treatment was effective, as the body weight of lizards decreased in the restricted but increased in the optimal food treatment (Fig. 1a). Temperature treatment or SVL did not affect the body weight change during the experimental period (Table 1). UV chroma expression was affected by the temperature treatment (Table 1). While UV chroma increased in every treatment group, it increased more steeply in the optimal temperature treatment (Fig. 1b). Temperature treatments had also an effect on UV brightness (Table 1). UV brightness showed a similar, albeit weaker trend than UV chroma (Fig. 1b, c). Neither UV chroma nor UV brightness change were affected by the food treatment or SVL (Table 1). None of the treatments affected blue chroma, blue brightness (Table 1, Fig. 1d,e) or total brightness (Table 1). The positive change of UV reflectance (in both absolute and relative terms) in all treatment groups is expected in the period during the experiment. In nature, nuptial colour becomes more and more pronounced after hibernation until it reaches its peak intensity during the reproductive period.

**Table 1:** Results from the General Linear Models on the body weight and throat colour change of male European green lizards during the experiment.

	Body weight	Total brightness	UV chroma	UV brightness	Blue chroma	Blue brightness
Food (F)	15.92**	0.02	1.02	0.12	1.11	0.24
Temperature (T)	1.71	2.08	5.09*	4.26*	1.36	3.26§
SVL	0.68	3.01	<0.01	1.43	1.05	1.16
F×T	<0.001	0.46	0.09	0.56	0.05	0.61

*F* values (*df*s = 1, 52) are shown.

For the descriptions of the models, see Material and Methods.

**P<0.001;

*P<0.05;

§ *P*<0.1.

## Discussion

Our most salient finding is that the development of the structural nuptial UV colour (both UV brightness and chroma) in male *L. viridis* – that is presumably under positive sexual selection [36], [38] – is affected by the time spent at high temperature. Interestingly, food availability that affected body condition had no effect on the UV colour expression of male *L. viridis*. On the other hand, time available for maintaining high body temperature was a strong predictor of the magnitude of increase in UV reflectance (both in absolute and relative terms) during the pre-mating period, even though it did not influence body condition. It is also noteworthy that the temperature treatment did not affect total brightness or blue brightness, only the UV range, further supporting the role of UV as a separate signal.

To our knowledge, there is no data on the exact mechanism how temperature might affect structural colour development in ectotherms. However, considering that this nuptial structural colour is a dynamic signal (as it fades in-and-out of peak intensity in every mating period), it might be affected by environmental factors [51]. Structural colour depends on the multitude and structural precision of the light scattering structures. Environmental stress may perturb the biochemical reactions during which these structures are produced. Thus, it might affect structural colour development as it has been showed in butterflies [31]. From a mechanistic point of view several scenarios are possible. Short-term colour change can be explained by nanostructure movements within the cells of the light reflecting layer [11], and similar, but longer-term effects might be possible. Alternatively, the synthesis of melanin, a pigment known to often underline structural colour and act as a purifier layer [52], can be temperature-dependent and down regulated by low body temperature, causing less intensive structural colouration. Finally, developmental stability can be temperature dependent in ectotherms [53], and thus suboptimal temperature might cause subpar seasonal colour development even in adults.

Theory predicts that sexual signals can be honest if they are costly for the signaller, and their cost is correlated with the signaller's quality [7], [8]. Further, according to the genic capture hypothesis, heritability of body condition and condition-dependence of the sexually selected signal are key mechanisms of maintaining costly sexual signals [4], [8], [54]–[59]. Hence, energy allocation to sexual signals seems to be common in both direct and indirect ways [60]–[62]. As sexual signals become costly, condition-dependence is expected to evolve, because only individuals of higher condition are able to pay the higher marginal costs of bearing exaggerated signals [58]. For instance, carotenoid- and melanin-based [63]–[65], and structural colours [66]–[69] can all act as condition-dependent signals both in mate choice and male dominance [70]–[73]. However, not only energetic costs and metabolic constrains can affect the development and maintenance of sexually selected signals, and condition-dependence is not always present. For instance, there are traits signalling hormone levels [74], immune activity [75], developmental stability [76], [77], oxidative balance [78], parasite load [79], [80] and overall health of the individual [80], [81] which are all important descriptors of the physiological state that affect both reproductive success and survival [82]. There are also examples of environmental factors affecting structural colours. For instance, manipulation of larval resource acquisition (host plant quality and low/high temperature shocks during metamorphosis) has a significant effect on male dorsal colouration in a butterfly species [31], [32]. Our results add a new example to the body of work suggesting that there are sexually selected colour traits that lack general condition-dependence, and signal other aspects of physiology [83], [84].

Ectotherms can survive and function with a wider range of body temperature than endotherms, however, their physiological performance may also vary more intensively with temperature [43]. Hence, we suggest that development of the structural nuptial colour in male *L. viridis* depends on time available for high physiological performance, which is directly affected by ambient temperature, but not by available energy. Further, optimal body temperature is mainly achieved by means of behavioural thermoregulation in small bodied ectotherms [85], which is costly in terms of time and energy invested, and also due to increased predation risk and lost opportunities of feeding and mating [46], [86]. Hence, the thermoregulatory cost of maintaining high body temperature and thus high physiological performance – especially early in the season for this temperate zone reptile – can confer reliability to UV signals on male *L. viridis*.

In a correlative study based on a three year dataset, we found a negative trend between body condition and UV chroma in male European green lizards during the mating season (Molnár O Bajer K, Török J, Herczeg G unpublished data). This result suggests that developing, maintaining and/or wearing intense UV throat colour has direct and/or indirect costs for the signaller. Our present results rejected the hypothesis that developing the UV colour has direct energy needs, and provide one possible pathway of how UV development can be costly via the importance of accurate and effective behavioural thermoregulation. Obviously, other possible costs like elevated level of aggression from conspecifics for instance should be studied in the future to fully understand the information content of this signal. Nor the above mentioned correlative study, neither the present experiment revealed any correlation between body size (an age proxy in lizards with indeterminate growth) and UV colour, suggesting that UV can signal age-independent individual quality. It is also possible, that UV throat colour acts as a multicomponent signal, in which the size of the patch refers to the body/head size, and UV colour (UV brightness or chroma) refers to the physiology of the bearer. That context would explain the lack of covariance between body size and nuptial throat colour. However, it is unknown if there is an allometric relationship between patch size and body/head size.

Taken together, the effect of ambient temperature on the development of a sexually selected structural colour trait in our study implicates that signals advertising physiological state of an ectotherm through a “temperature-physiology-trait” pathway can be of comparable importance to condition-dependent traits advertising nutritional condition through a “food-body condition-trait” pathway. Our results offer a possible environmental constraint on and reveal the possible costs of a sexually selected structural colour signal in a reptile. Nevertheless, we provide an example that not only the available nutrients can be limiting in developing a sexual colour signal (as it has been shown in previous studies). Losing body weight in the high temperature/low food treatment did not stop male *L. viridis* from developing their UV signal more than males in the low temperature treatments, irrespective of food. This suggests that structural colour development in *L. viridis* requires little energy and/or nutrients, and it may be affected by other aspects of physiology. On the other hand, despite *ad libitum* food, male lizards failed to develop their signal maximally without optimal access to heat. Future studies on this system should aim to reveal (i) what aspect of physiology is signalled through nuptial UV colouration, (ii) if melanin plays a role in sexual signalling, (iii) if there are other colour traits or trait combinations that predict the variation in body condition and (iv), if UV development and spring weather are connected in nature.
